# Integrated pyroptosis measurement and metabolomics to elucidate the effect and mechanism of tangzhiqing on atherosclerosis

**DOI:** 10.3389/fphys.2022.937737

**Published:** 2022-09-12

**Authors:** Rui Chen, Ting Chen, Zhihuan Zhou, Zhihui Song, Wanying Feng, Xintong Yang, Xianliang Wang, Bin Li, Xinya Ding, Han Zhang, Yi Wang

**Affiliations:** ^1^ College of Traditional Chinese Medicine, Tianjin University of Traditional Chinese Medicine, Tianjin, China; ^2^ Key Laboratory of Hunan Province for Integrated Traditional Chinese and Western Medicine on Prevention and Treatment of Cardio-Cerebral Diseases, Hunan University of Chinese Medicine, Changsha, China; ^3^ Institute of Traditional Chinese medicine, Tianjin University of Traditional Chinese Medicine, Tianjin, China; ^4^ National Clinical Research Center for Chinese Medicine Acupuncture and Moxibustion, First Teaching Hospital of Tianjin University of Traditional Chinese Medicine, Tianjin, China; ^5^ Tianjin State Key Laboratory of Modern Chinese Medicine, Tianjin University of Traditional Chinese Medicine, Tianjin, China

**Keywords:** tangzhiqing, inflammasomes, pyroptosis, atherosclerosis, Chinese traditional medicine

## Abstract

Tangzhiqing formula (TZQ) is a traditional Chinese medicine prescribed to treat glucose and lipid metabolism disorders. A significant effect of TZQ on diabetes and hyperlipidemia has been demonstrated, but its effect on atherosclerosis (AS) remains unknown. This study combines pyroptosis with metabolomics to elucidate the effect and mechanism of TZQ on AS. A model of AS was developed using ApoE^−/−^ mice fed a high-fat diet for 8 weeks. After 6 weeks of atorvastatin (Ator) or TZQ treatment, aortic lumen diameter, aortic lesion size, serum lipid profile, cytokines, and Nod-like receptor protein 3 (NLRP3) inflammasome-mediated pyroptosis were analyzed. Serum metabolomics profiles were obtained to examine the effect of TZQ on AS and the correlation between pyroptosis and metabolites was further analyzed. As a result, TZQ significantly reduced the diameter of the common carotid artery during diastole and the blood flow velocity in the aorta during systole; reduced blood lipid levels, arterial vascular plaques, and the release of inflammatory cytokines; and inhibited the NLRP3 inflammasome-mediated pyroptosis. According to metabolomics profiling, TZQ is engaged in the treatment of AS *via* altering arachidonic acid metabolism, glycerophospholipid metabolism, steroid hormone production, and unsaturated fatty acid biosynthesis. The cytochrome P450 enzyme family and cyclooxygenase 2 (COX-2) are two major metabolic enzymes associated with pyroptosis.

## 1 Introduction

AS is the main pathological basis and contributor to cardiovascular diseases (Tabas et al., 2015). Additionally, AS is a chronic inflammatory condition: inflammation contributes both to the development and maintenance of the disease (Geovanini and Libby, 2018). Another important pathological mechanism of AS is cell death. A type of cell death called Pyroptosis is followed by an inflammatory response controlled by inflammasomes. The inflammasome NLRP3 plays a crucial role in AS. When NLRP3 is activated, its N-terminal pyrin domain (PYD) interacts with and activates the downstream ASC [apoptotic speckle-like protein containing a caspase recruitment domain (CARD)] protein. ASC, in turn, dimerizes and hydrolyzes pro-caspase-1 N-terminal CARD domains to generate mature caspase-1. caspase-1 converts immature interleukin (IL)-18 and IL-1β into mature inflammatory mediators. Additionally, it cleaves the gasdermin protein family member D (GSDMD), creating GSDMD-N fragments that oligomerize, promote membrane pores, and stimulate the release of inflammatory chemicals, resulting in cell swelling and pyroptosis (Schroder and Tschopp, 2010).

There is evidence that pyroptosis plays an important role in the development and progression of AS. It's been found that AS plaques overexpress NLRP3 and caspase-1. Activated inflammasomes cause the release of IL-1β and IL-18, resulting in an inflammatory cascade. In ApoE^−/−^ mice, inhibiting the NLRP3 inflammasome decreases atherosclerotic plaques (Menu et al., 2011). The AS plaques of ApoE^−/−^ and IL-1β^−/−^ mice were decreased approximately 30% when compared with ApoE^−/−^ mice, demonstrating that IL-1β is a key role in the development of AS (Gomez et al., 2018). Ascending aortic tissue from coronary artery bypass grafting patients showed significantly higher levels of NLRP3 expression than those without AS (Zheng et al., 2013). When compared with healthy arteries, those with carotid plaques had considerably higher transcriptional and protein levels of NLRP3, ASC, and caspase-1, as well as IL-1β and IL-18 (Shi et al., 2015). As a result of caspase-1 activity, endothelial cells are activated via IL-1β/IL-18-dependent mechanisms, aggravating the inflammatory response and causing an intensification of AS lesions (Tang et al., 2019). As endothelial cells are inhibited by caspase-1, there is a reduction in AS development and pyroptosis in endothelial cells (Yang et al., 2019). In conclusion, inflammation, NLRP3/caspase-1 and other pyroptosis-related inflammasome proteins play an important role in the development of AS.

Arachidonic acid produces epoxyeicosatrienoic acids (EETs), important blood vasodilators, via cytochrome P450 enzymes. Consequently, cytochrome 450 enzymes have been demonstrated to enhance reactive oxygen species (ROS) generation, hyperlipidemia, and AS. For example, CYP1B1 has been observed to promote ROS production, hyperlipidemia, and AS (Song et al., 2016). Overexpression of CYP2J2 enhances EET levels in ApoE^−/−^ mice and protects against AS caused by a high-fat diet (Liu et al., 2016). Likewise, CYP7A1 has a preventive function against AS, and overexpression of CYP7A1 reduces AS by increasing bile secretion, decreasing serum low density lipoprotein C (LDL-C), and reducing visceral fat adaptation (Krishnamurthy et al., 2016). CYP4A11 has anti-atherosclerotic properties (Fu et al., 2013). Conversely, patients with CYP2C9 mutant alleles have a lower metabolic capacity, which leads to lower endothelium-derived hyperpolarizing factor production and a higher risk of AS (Ercan et al., 2008). AS plaque stability can be significantly influenced by COX-2, an enzyme that catalyzes the metabolism of arachidonic acid (Cipollone and Fazia, 2006). Growing evidence suggests that metabolomics can serve as an efficient approach to discover new biomarkers of AS disease and the pathogenic mechanism of AS can be clearly revealed by analyzing metabolic states (Blake et al., 2002). Metabolites interfere with the progress of AS by interacting with certain inflammasome proteins and mediators of cell pyroptosis during the development of AS.

TZQ is a traditional Chinese herbal medicine prescription that can effectively treat a variety of diseases. It is mainly composed of *Morus alba* L., *Nelumbo nucifera* Gaertn, *Crataegus pinnatifida* Bunge var. *major* N. E. Br, *Salvia miltiorrhiza* Bungege, and *Paeonia lactiflora* Pall. TZQ, also known as Sanye Tablet, is an effective formula for the treatment of glucose and lipid metabolic abnormalities that may also be used to treat a variety of ailments. Based on chemical and metabolic analyses of TZQ disposition, 86 components and 48 metabolites were detected in rat plasma, urine, and feces. The primary components absorbed are flavonoids and alkaloids, while the primary metabolites are glucuronides and sulfates (Wu et al., 2018b).

We previously demonstrated that TZQ significantly reduced serum cholesterol (TC), triglycerides (TG), low density lipoprotein cholesterol (LDL-C), and liver triglyceride and TC levels in type 2 diabetic rats, as well as increased high-density lipoprotein C (HDL-C)/TC levels in hyperlipidemic rats. When TZQ is administered to hyperlipidemic rabbits, watery degeneration and fatty degeneration of their livers are significantly reduced, as well as inflammatory cell infiltration (Zhanbiao et al., 2011b) and significantly improves myocardial histopathological changes, cardiac function, and structural changes in obese mice (Yao et al., 2021). Furthermore, TZQ minimizes aortic intima damage, lipid infiltration, and vascular occlusion, and has a protective impact on endothelial injury in the early stages of hypercholesterolemia-induced aortic AS. (Zhanbiao et al., 2011a; Liu et al., 2018). There has been increasing evidence that inflammation and the NLRP3/caspase-1 inflammasome play a crucial part in the formation of AS; for example, plaque growth was significantly attenuated in ApoE^−/−^ mice when NLRP3 was inhibited (Li et al., 2021). Metabolomics research has discovered that TZQ relieves the clinical signs of type 2 diabetes and hypertriglyceridemia patients by modulating glycerophospholipid metabolism (Liu et al., 2018).

The precise mechanism of TZQ’s anti-atherogenic effects is unclear. Therefore, we hypothesized that TZQ has an anti-atherosclerotic effect through inhibiting NLRP3-mediated pyroptosis and employed serum metabolomics to investigate the metabolites through which TZQ operates on the inflammasome and pyroptosis to interfere with AS.

## 2 Materials and Methods

### 2.1 Animals

Seventy-two 8-week-old male ApoE^−/−^ mice and ten wild-type C57BL/6J mice weighing (20 ± 2 g) were used in this study. The mice were obtained from SPF (Beijing) Biotechnology Co., Ltd. and the certificate number was SCXK (Beijing) 2019-0010. All animals were maintained under conditions of temperature (20 ± 5°C), relative humidity (55 ± 5%), alternating lighting (12 h light/12 h dark cycle) and free access to diet. All the experimental protocols were conducted in accordance with the guidelines approved by the Animal Care Committee of Tianjin University of Traditional Chinese Medicine and Animal Ethical Committee of Tianjin University of Traditional Chinese Medicine (TCM–LAEC2021037).

### 2.2 Preparation of medicines

TZQ was provided by the Department of Pharmacy, Institute of Traditional Chinese Medicine, Tianjin University of Traditional Chinese Medicine. After soaking the above five traditional Chinese medicines in water, they were decocted twice, filtered, and combined; the filtrate was concentrated into a thick paste, dried under reduced pressure, and crushed into powder. *Morus alba* L. 666.7 g and *Nelumbo nucifera* Gaertn 666.7 g were extracted with 50% ethanol and water and concentrated into a dry cream. *Crataegus pinnatifida* Bunge var. *major* N. E. Br 666.7 g and *Salvia miltiorrhiza* Bungege 833.3 g are extracted with 70% and 50% ethanol respectively, adsorbed on D101 macroporous resin, eluted with 70% ethanol and concentrated into dry powder, *Paeonia lactiflora* Pall 833.3g is extracted with water, eluted with 70% ethanol and concentrated into dry powder. The drug ratio of the ointment was as follows: Morus alba L: 19.75%; Nelumbo nucifera Gaertn: 19.75%; Crataegus pinnatifida Bunge var. major N. E. Br: 6.25%; Salvia miltiorrhiza Bunge: 6.3%; and Paeonia lactiflora Pall: 4.7%. TZQ was found to contain lotus alkaloid, paeoniflorin, salvianolic acid B, hypericin, and rutin (6.40, 1.75, 1.70, 0.004, and 0.006 mg, respectively) (Li et al., 2018). In another study, the concentrations of five components in TZQ were found to be as follows: chlorogenic acid: 7.859–19.93 mg L^−1^, paeoniflorin: 127.7–405.7 mg L^−1^, rutin: 6.505–13.51 mg L^−1^, Hyperoside: 6.856–39.61 mg L^−1^, quercetin-3-O-β-D-glucuronide: 56.30–208.7 mg L^−1^; and salvianolic acid B: 82.60–564.0 mg L^−1^ (Shao et al., 2019). Atorvastatin calcium (Batch no. J20070060) was produced by Pfizer Inc. (New York, NY, United States). Sodium nitroprusside (SNP) (Batch no. 71778-25G) was purchased from Sigma–Aldrich (St. Louis, MO, United States).

### 2.3 Grouping and drug administration

Before the experiments, all animals were fed a normal diet for 1 week, the C57BL/6J mice in the control group (Control) were fed a normal chow diet, ApoE^−/−^mice were given a high-fat diet for 8 weeks (MD12015A, Medicience, China) to create an AS model. As soon as the model was successful, ApoE^−/−^mice were randomly separated into four groups based on blood lipids, including a model group (Model), a TZQ (4 g/kg) group, a TZQ (8 g/kg) group, and a positive drug control group [Atorvastatin (Ator) 3 mg/kg] with *n* = 18 mice per group. TZQ was administered by gavage and its vehicle [0.5% carboxymethylcellulose sodium (CMC–Na)] was administered to the Control and Model groups. Each group was administered comparable medicines at a dose of 10 ml/kg, once a day for 6 weeks.

### 2.4 Aortic blood velocity and lumen diameter of the common carotid artery

The neck and chest hair of each mouse was shaved. The mice were placed supine on a constant temperature examination table, the temperature was kept at 37°C, the heart rate was maintained at 300–400 beats/min, and the medical couplant was applied to the chest for ultrasonic examination (Vevo2100, FUJIFILM VisualSonics, Canada). The probe was parallel to the long axis of the mouse, close to the sternum of the mouse, and maintained at a 30–45° angle with the chest wall of the mouse. The direction knob was adjusted, and the ascending aorta long axis, the aortic arch and its branches, and the long axis of the descending aorta were obtained under B-Mode. The probe to the left side of the neck was tilted and the head was moved gently to clearly reveal the branches of the carotid artery. The instrument was switched to color mode and the blood flow rate at the aortic arch was measured. The dynamic images of the aortic arch and sections of the strong arteries were collected continuously for 10 s. After storing the images, the image analysis system (VisualSonics, Toronto, ON, Canada) was employed for offline analysis of the dynamic images of the aortic arch and sections of the strong arteries.

### 2.5 Quantification of lipid levels in the blood and fat mass

After the administration, blood was collected from the medial canthus of all mice, and after standing for 30 min, the serum was separated by centrifugation at 3500 rpm and 4°C for 10 min (Legend Micro 17, Thermo Scientific, Waltham, MA, United States). The levels of TC, TG, LDL-C and HDL-C in serum were determined with an automatic biochemical analyzer (Microlab300, Vertu, Netherlands). The kidneys and testes of ApoE^−/−^ mice were isolated; the perirenal and peritestiscular fat was dissected, the wet weight was measured (JA1003, Shanghai HengPing Instrument and Meter Co., Ltd., China), and the fat coefficient was calculated (fat wet weight/body weight × 100%).

### 2.6 Arterial plaque and collagen content

Intact aortas of each group of mice were dissected and isolated by microscopy (Leica S8 APO, Leica Microsystems, Wetzlar, Germany), removed external fatty deposits stripped. The aorta was placed in 4% paraformaldehyde solution (P1110, Solarbio, China) followed by 70% isopropanol (8018GR0500, Tianjin Xiehe, China), stained with Oil Red O staining solution (G1261, Beijing Solarbio, China), rinsed with distilled water, and photographed.

#### 2.6.1 Oil Red O staining

Aortic roots were fixed, dehydrated, and cryosectioned (RM 2016, Leica Microsystems). The thickness of the slices was 10 μm. After staining with Oil Red O staining solution for 10 min, the slices were soaked in 60% isopropanol for 30 s, and then stained with hematoxylin for 2 min. After Oil Red O staining, images of the sections were collected using a digital microscope (Pannoramic MIDI, 3D HIES TECH, Hungary) and plaque area analysis was performed with the Image-ProPlus 6.0 software program.

#### 2.6.2 Haematoxylin-Eosin Staining

Aortic tissue was fixed in 4% paraformaldehyde, then embedded in paraffin, and paraffin sections (5 μm) were cut and mounted on glass slides for haematoxylin-eosin (HE) staining. Hematoxylin solution was stained for 3 min and eosin solution for 90 s images of the sections were collected using a digital microscope (Pannoramic MIDI, 3D HIES TECH, Hungary) and plaque area analysis was performed with the Image-ProPlus 6.0 software program.

#### 2.6.3 Masson Staining

Aortic root sections were fixed in 4% paraformaldehyde, then embedded in paraffin, and paraffin sections (5 μm) were cut and mounted on glass slides for masson staining, the paraffin sections were deparaffinized with xylene solution, dehydrated with graded ethanol solutions, and stained according to the procedure of the Masson staining kit (G1346, Beijing Solarbio). Images of the sections were collected using a digital microscope (Pannoramic MIDI, 3D HIES TECH, Hungary) and collagen analysis was performed with the Image-ProPlus 6.0 software program.

### 2.7 Inflammatory response

The levels of TNF-α, IL-6, IL-1β, and IL-18 inflammatory factors in ApoE^−/-^ mice serum were measured with enzyme-linked immunosorbent assays to evaluate the effect of TZQ on the inflammatory response using the specific experimental methods recommended by the manufacturer (Boster Biological Technology, Pleasanton, CA, United States). Color development at 450 nm was then measured using an ELISA autoanalyzer (Enpire, PERKin Elmer, United States).

### 2.8 Immunofluorescence analysis

The expression of NLRP3/caspase-1 in the aortic root was detected with an immunofluorescence method. The frozen sections of the aortic root were placed in 0.5% TritonX-100 solution (E-IR-R122, Elabscience, Wuhan, China) for 20 min, blocked with goat serum (C0265, Beyotime, Shanghai, China), for 30 min, incubated with the primary antibody NLRP3 (1:100 dilution, Proteintech, United States), caspase-1 (1:100 dilution, Proteintech, United States) at 4°C overnight in a refrigerator, soaked in phosphate buffered saline-Tween 20 (PBST) wash, incubated with the secondary antibodies goat anti-rabbit IgG (Alexa Fluor^®^ Plus 594, A32740, Invitrogen, Carlsbad, CA, United States) or goat anti-mouse IgG (Alexa Fluor^®^ Plus 488, A32723, Invitrogen), at 37°C for 2 h, incubated with 4′,6-diamidino-2-phenylindole (DAPI), at 37°C for 5 min and imaged using a fluorescence microscope (CKX41-F32FL, Olympus, Japan).

### 2.9 Real-time quantitative PCR analysis

Total RNA was extracted from the aortas from the ApoE^−/−^ mice with TRIzol (Life Technologies, Carlsbad, CA, United States). After the aortas were fully lysed and centrifuged, the RNA pellet was washed, dried, and quantified (NanoDrop™ One, Thermo Scientific, Waltham, MA, United States). RNA was reverse transcribed using the 5x HiFiScript RTMaster Mix kit as directed by the manufacturer (18091200, Cowin Biotech, Jiansu, China). The MagicSYBR Mixture was used for amplification according to the manufacturer’s instructions (CW0659, CoWin Biosciences). The primers are listed in Table 1. The results were analyzed by the 2^−ΔΔCT^ method with glyceraldehyde-3-phosphate dehydrogenase (GAPDH) as the internal control.

**TABLE 1:** The primer sequences.

Gene	Primer sequence
NLRP3	F: GCC​GTC​TAC​GTC​TTC​TTC​CTT​TCC
	R: CAT​CCG​CAG​CCA​GTG​AAC​AGA​G
Caspase-1	F: ATA​CAA​CCA​CTC​GTA​CAC​GTC​TTG​C
	R: TCC​TCC​AGC​AGC​AAC​TTC​ATT​TCT​C
ASC	F: GGA​CGG​AGT​GCT​GGA​TGC​TTT​G
	R: CAT​CTT​GTC​TTG​GCT​GGT​GGT​CTC
GSDMD	F: CGA​TGG​GAA​CAT​TCA​GGG​CAG​AG
	R: ACA​CAT​TCA​TGG​AGG​CAC​TGG​AAC

### 2.10 Western blot analysis

The aortic tissue from ApoE^−/−^ mice was fully lysed with protein lysis buffer, and the supernatant was collected using a bicinchoninic acid (BCA) kit according to the manufacturer’s instructions (PC0020, Beijing Solarbio). The samples were mixed with 5X loading buffer and boiled at 100°C for 10 min to denature proteins. The proteins were then separated on a 10% SDS-PAGE gel (80 V, 30 min, and then 120 V, 60 min) and transferred to PVDF membranes (350 mA, 90 min). After blocking with 5% (w/v) nonfat milk for 2 h, the membrane was washed twice with TBST buffer, 10 min/time (CW0043, CoWin Biosciences), incubated with the primary antibody overnight at 4°C, rinsed four times with TBST for 10 min/time. incubated with the secondary antibody for 2 h at room temperature, washed 4 times with TBST for 10 min/time, and the immunoreactive bands were visualized with enhanced chemiluminescence (ECL) reagent in the dark (WBULS0500, Millipore, MA, United States). Relative expression of proteins was analyzed using an image analysis system (Image J, United States). The antibodies included rabbit polyclonal NLRP3 (1:1,000 dilution, ProteinTech, Rosemont, IL, United States), rabbit polyclonal caspase-1 (1:1,000 dilution, Proteintech), mouse monoclonal GAPDH (1:50,000 dilution, Proteintech), mouse monoclonal ASC (1:1,000 dilution, Santa Cruz Biotechnology, Dallas, TX, United States), and mouse monoclonal GSDMD (1:500 dilution, Santa Cruz Biotechnology).

### 2.11 Metabonomic analysis

Acetonitrile (300 μL) was added to each 100 μL ApoE^−/−^ mice serum sample and the mixture was ultrasonicated for 10 min in an ice water bath, vortexed, and centrifuged for 15 min at 4°C at 1040 × g. The supernatant was collected and analyzed using the ACQUITY UPLC I-Class Ultra Performance Liquid Chromatography System (ACQUITY UPLC I, Waters Corporation, Milford, MA United States). The chromatographic peaks were separated at 30°C using a Waters ACQUITY UPLC BEH C18 (2.1 × 100 mm, 1.7 µm) chromatographic column with acetonitrile (B) –0.1% formic acid water (A) as the mobile phase at a flow rate of 0.4 ml/min. Other important parameters include the following: Elution: 0–9 min: 97%A–0%A; 9 min–10 min: 0%A–0%A; 10 min–10.1 min: 0%A–97%A; 10.1 min–12 min: 97%A. MS analysis was carried out on the Q Exactive™ Plus Combination Quadrupole Orbitrap™ Mass Spectrometer (IQLAAEGAAPFALGMBDK, Thermo Scientific, Waltham, MA, United States). The detection mode was positive and negative ion detection with parameter settings as follows: spray voltage–3.0 kV/+3.5 kV; sheath gas (N_2_) 35 L/h; auxiliary gas (N_2_) 10 L/h; purge gas (N_2_) 0 L/h; capillary temperature 350°C, and auxiliary gas heating temperature 350°C. The complete scan range was m/z 100–1,500, the resolution was 70,000, and the automatic gain value [automatic gain control (AGC) target] was set to 3e6. The MS 2 mass spectrum scan was the dynamic mass range, the resolution was 17,500, and the AGC was 1e5. At normalized collision energy (NCE) 20/40/60 V, collision-induced dissociation [higher energy collisional dissociation (HCD)] was carried out.

For multivariate statistical analysis, the data were entered to SIMCA14.0 software. The screened substances that contributed to the classification [variable importance in projection (VIP) score >1] were used as candidate metabolic biomarkers after performing unsupervised discriminant analysis with principal component analysis (PCA) followed by supervised partial least squares discriminant analysis (PLS-DA), and the screened substances that contributed to the classification (VIP > 1) were used as candidate metabolic biomarkers. Finally, substances with *p* < 0.05 were used as biomarkers of significant difference; mass numbers of markers (m/z value) and the HMDB (http://www.hmdb.ca/) and MetaboAnalyst5.0 (https://www.metaboanalyst.ca) databases were used to search for, identify, and confirm substances as possible metabolic biomarkers. The MetaboAnalyst 5.0 database and Cytoscape software were used to perform map and metabolic pathway analysis. To show evolutionary maps, a corresponding metabolic network was created using the Cytoscape software plug-in metscape.

### 2.12 Statistical analysis

SPSS 25.0 was used to analyze the data; the measurement data were expressed as the mean ± standard deviation and were analyzed by one-way analysis of variance (ANOVA). The enumeration data were analyzed by chi-square test. *p* < 0.05 was considered statistically significant. GraphPad Prism 8.0 software was used for data processing and graphing.

## 3 Results

### 3.1 Effects of tangzhiqing formula on aortic blood flow velocity and common carotid artery lumen diameter

AS is a disease that primarily affects the intimal layer of the arterial wall, but ultrasound imaging cannot distinguish the intimal and middle layers of the arterial wall. An increase of intima-media thickness of common carotid artery may reflect an increase of intima thickness. It is becoming more and more evident that increased intima-media thickness of the common carotid artery is a sign of AS. Furthermore, plaque is tightly connected to arterial blood flow velocity. Peak systolic velocity (PSV) and end-diastolic velocity (EDV) are not only related to the amount of bleeding in the plaque, but also related to the degree of carotid stenosis.

**FIGURE 1:**
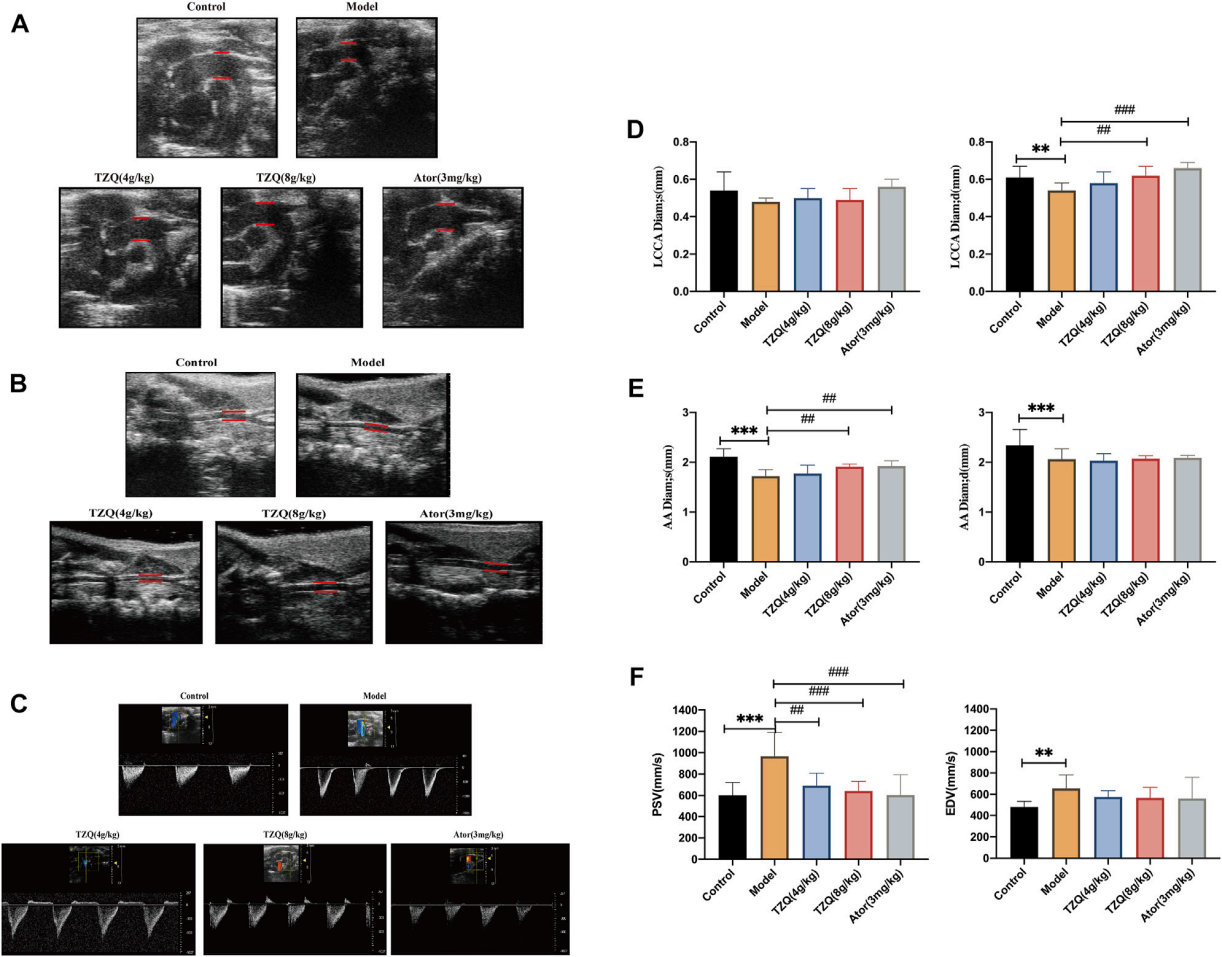
Aortic blood velocity and lumen diameter of common carotid artery. **(A)**: Ultrasound of the aortic arch. **(B)**: Carotid artery ultrasound. **(C)**: Aortic flow ultrasonography. **(D)**: Systolic and diastolic distal diameters of the common carotid artery. **(E)**: Aortic systolic and diastolic diameters. **(F)**: Aortic arch blood flow velocities systolic and diastolic. LCCA Diam;s: Distal systolic diameter of common carotid artery; LCCA Diam;d: Distal common carotid artery diastolic diameter; AADiam;s: Aortic systolic diameter; AADiam;d: Aortic diastolic diameter; PSV: Aortic arch systolic blood flow velocity; EDV: Aortic arch diastolic blood flow velocity. *n* = 13 mice for each group, **p < 0.05 versus Control, ##*p* < 0.05, ###*p* < 0.01 versus Model.

The ultrasound results are displayed in (Figures 1A,B,D,E). Compared with the Control group, the left common carotid artery (LCCA) and ascending aorta (AA) in the Model group had significantly higher blood vessel diameters during diastole and systole (*p* < 0.05) and blood flow velocity of the aortic arch than those of the Control group. Both systolic and diastolic periods were significantly increased in the model group (*p* < 0.01) (Figures 1C,F). Compared with mice in the Model group, mice in the TZQ 4 g/kg, TZQ 8 g/kg, and Ator 3 mg/kg groups had significantly lower blood flow velocity of the aortic arch (Figure 1F) during the systolic phase (*p* < 0.01), and the diameter of common carotid artery in diastolic phase was significantly lower (*p* < 0.01) (Figure 1F).

### 3.2 Effect of tangzhiqing formula on lipid metabolism

**FIGURE 2:**
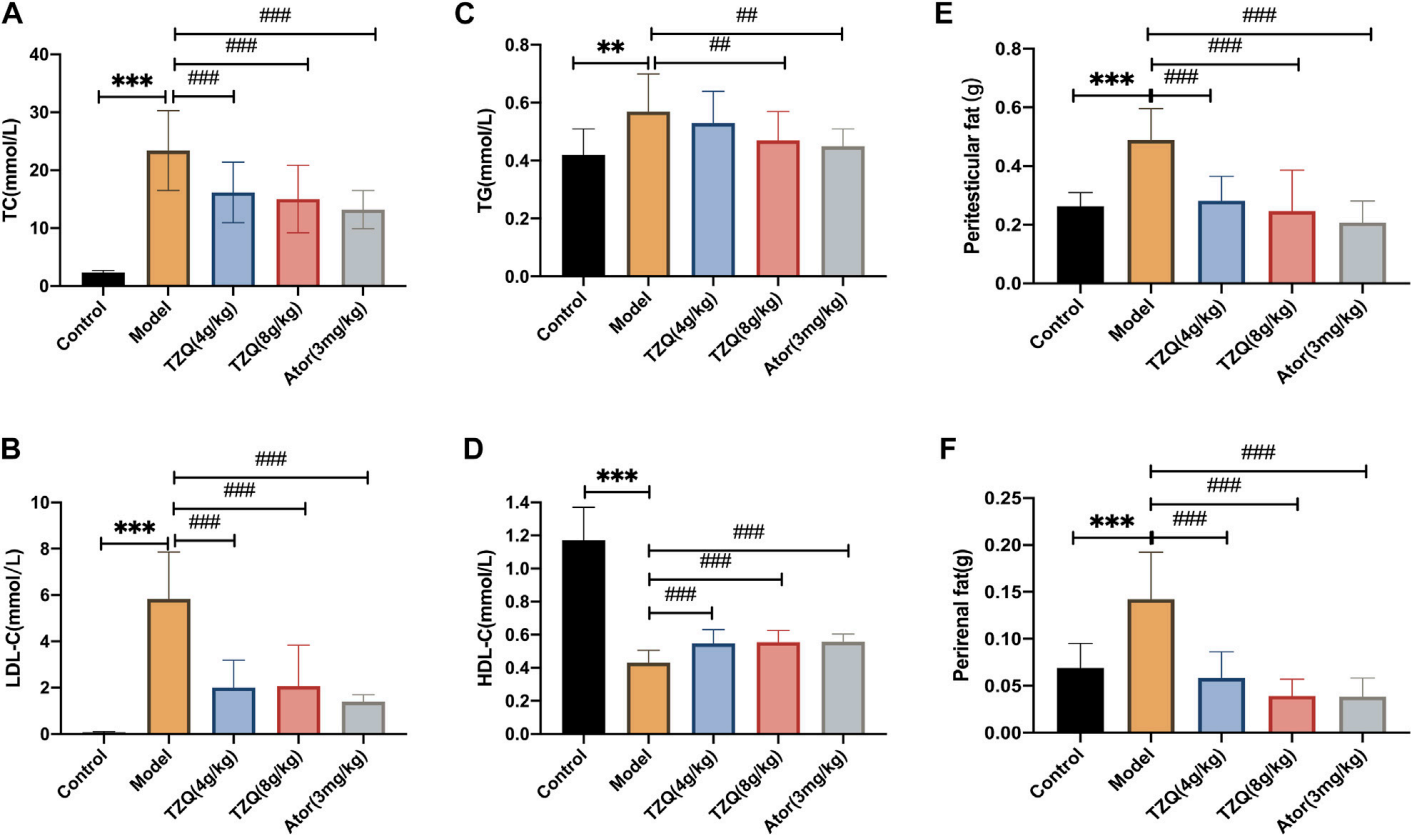
Effect of Tangzhiqing (TZQ) on lipid metabolism. **(A)**: TC. **(B)**: LDL-C. **(C)**: TG. **(D)**: HDL-C. **(E)**: Peritesticular fat. **(F)**: Perirenal fat. *n* = 13 mice for each group, **p < 0.05, ***p < 0.01 versus Control, ##*p* < 0.05, ###*p* < 0.01 versus Model.

Compared with those in mice in the Control group, the concentrations of TC, TG, and LDL-C in mice in the Model group were significantly higher (*p* < 0.01) (Figures 2A–C), and the masses of peritesticular and perirenal fat were significantly higher (*p* < 0.01) (Figures 2E,F), whereas HDL-C was considerably lower (*p* < 0.01) (Figure 2D). Compared with those in mice in the Model group, the concentrations of TC, TG, and LDL-C in mice in the TZQ 4 g/kg and Ator 3 mg/kg groups were significantly lower (*p* < 0.05), as was the amount of peritesticular and perirenal fat (*p* < 0.01). HDL-C concentrations were considerably elevated (*p* < 0.01).

### 3.3 Effect of tangzhiqing formula on aortic plaque

**FIGURE 3:**
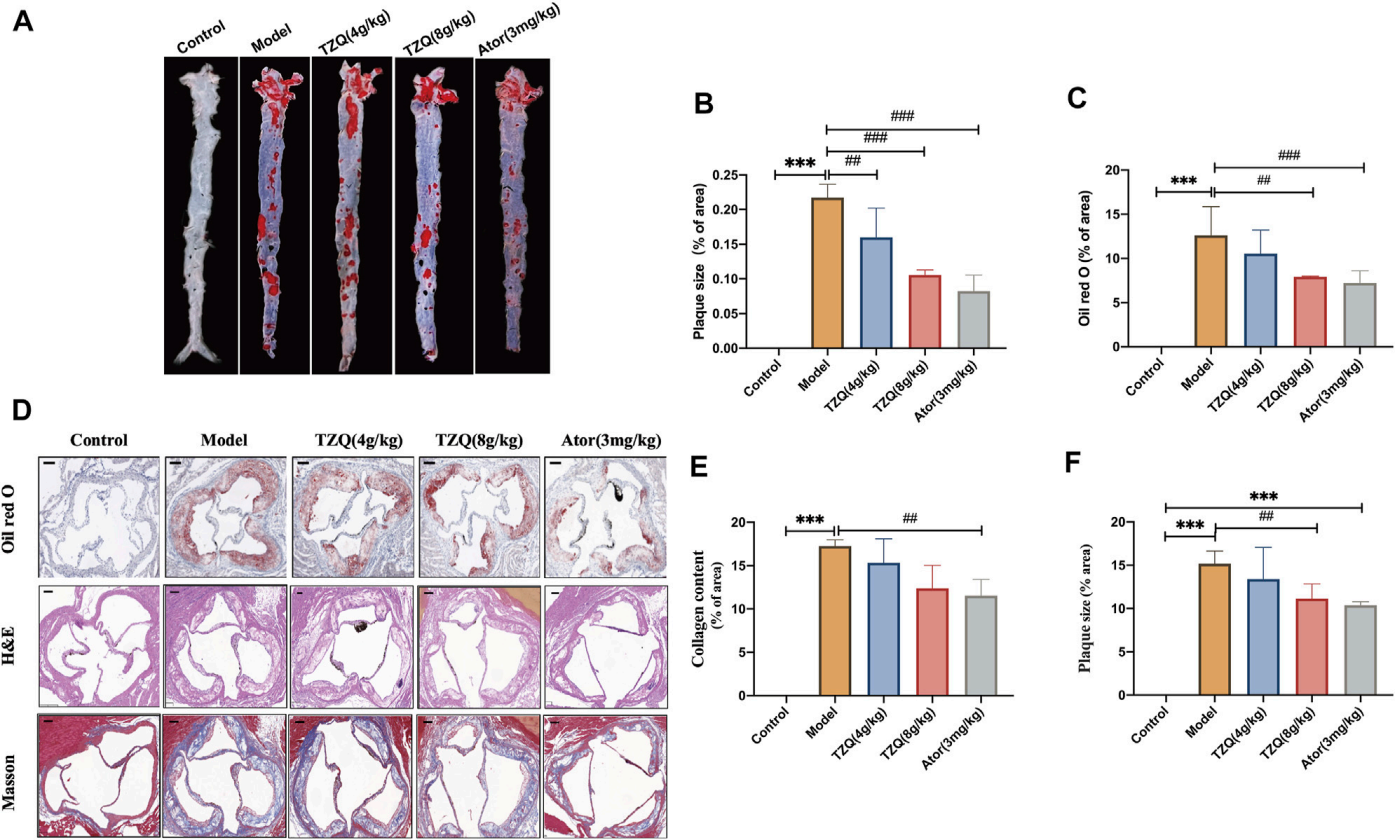
Effect of TZQ on aortic plaques. **(A)**: Gross Oil Red O staining of the aorta. **(B)**: Gross aortic plaque area. **(C)**: Aortic root plaque area. **(D)**: Oil Red O staining. **(E)**: Masson staining (×200), **(F)**: Hematoxylin staining (×200). Scale bar = 50 μm, *n* = 4 mice for each group, **p < 0.05, ***p < 0.01 versus Control, ##*p* < 0.05, ###*p* < 0.01 versus Model.

The aorta was stained with Oil Red O (Figures 3A,B). Compared with that in mice in the Control group, the aortic red plaque area in mice in the Model group was significantly higher (*p* < 0.01). Compared with that in mice in the Model group, the red plaque area was significantly reduced in mice in the TZQ 4 g/kg, TZQ 8 g/kg, and Ator 3 mg/kg groups (*p* < 0.01). It was discovered that both TZQ and Ator may considerably diminish the pathological staining of the aortic root (Figure 3D). Compared with those in mice in the Control group, the intima in mice in the Model group was significantly thicker, the blood vessels were significantly narrowed, and lipid deposition, red plaque (Figure 3C), plaque area (Figure 3F), and fibrosis (Figure 3E) were significantly higher (*p* < 0.01). Compared with those in mice in the Model group, the red plaque area and lipid deposition in mice in the TZQ 8 g/kg group were significantly lower (*p* < 0.01), and the degree of fibrosis was considerably lower (*p* < 0.01). Lipid deposition and fibrosis were also significantly lower in mice in the Ator 3 mg/kg group (*p* < 0.01).

### 3.4 Effect of tangzhiqing formula on inflammation

**FIGURE 4:**
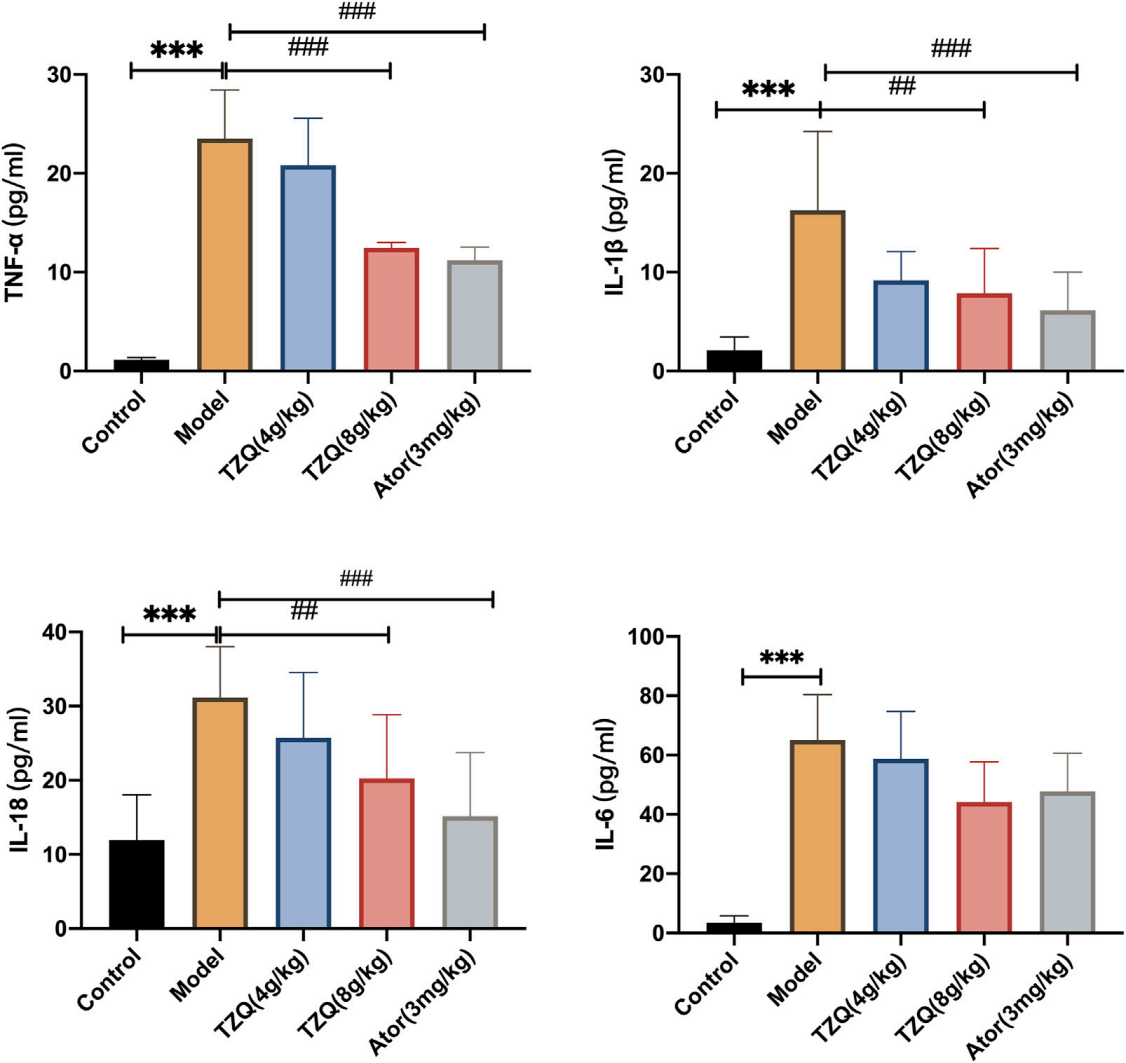
Effect of TZQ on inflammation. *n* = 4 mice for each group, **p < 0.05, ***p < 0.01 versus Control, ##*p* < 0.05, ###*p* < 0.01 versus Model.

AS is a chronic inflammatory disease in which inflammation is present at all phases. Compared with those in Control mice (Figure 4), serum levels of TNF-ɑ, IL-6, IL-18, and IL-1β were significantly higher in ApoE^−/−^ mice (*p* < 0.01). However, mice in both the TZQ 8 g/kg and Ator 3 mg/kg groups had significantly lower serum levels of TNF-ɑ, IL-6, IL-18, and IL-1β than those in ApoE^−/−^ mice (*p* < 0.05).

### 3.5 Effects of tangzhiqing formula on Pyroptosis

**FIGURE 5:**
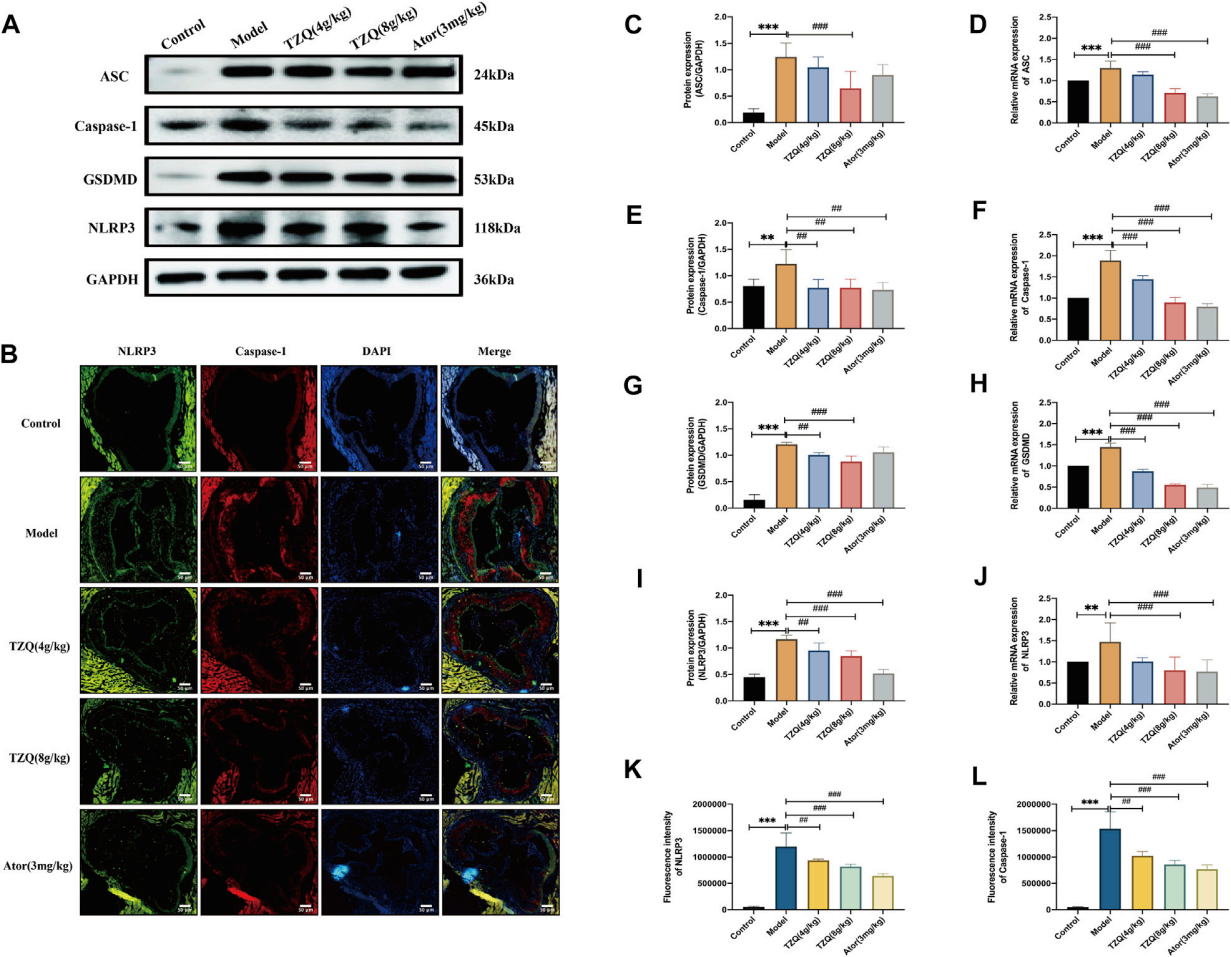
Effect of TZQ on pyroptosis. **(A)**: Western blot of pyroptosis-related proteins in the aortic root. **(B)**: Colocalization of Nod-like receptor protein 3 (NLRP3)/caspase-1 immunofluorescence in aortic root (×200). **(C,E,G,I)**: Quantitative analysis of gray values of bands from Western blots, GAPDH was used as loading control. **(D,F,H,J)**: Quantitative analysis of mRNA from RT-PCR. **(C)**: ASC. **(D)**: ASC mRNA. **(E)**: Caspase-1. **(F)**: Caspase-1 mRNA. **(G)**: Gasdermin protein family member D (GSDMD). **(H)**: GSDMD mRNA. **(I)**: NLRP3. **(J)**: NLRP3 mRNA. **(K)**: NLRP3 fluorescence density. **(L)**: Caspase-1 fluorescence density. Scale bar = 50 μm, *n* = 4 mice for each group, **p < 0.05, ***p < 0.01 versus Control, ##*p* < 0.05, ###*p* < 0.01 versus Model.

Compared with Control mice, the protein (Figures 5A,C,E,G,I) and gene (Figures 5D,F,H,J) expressions of NLRP3, caspase-1, ASC, and GSDMD in the aortic tissue of ApoE^−/-^ mice were significantly higher (*p* < 0.01). The fluorescence densities of NLRP3 and caspase-1 (Figures 5B,K,L) were dramatically higher (*p* < 0.01). However, treatment with TZQ 4 g/kg, TZQ 8 g/kg, and Ator 3 mg/kg substantially reduced NLRP3, caspase-1, and GSDMD protein expression (*p* < 0.05). Moreover, treatment with TZQ 8 g/kg and Ator 3 mg/kg significantly reduced NLRP3, caspase-1, ASC, and GSDMD mRNA expression (*p* < 0.01), as well as the fluorescence densities of NLRP3 and caspase-1 (*p* < 0.01).

### 3.6 Tangzhiqing formula metabolic biomarker screening in the treatment of atherosclerosis

**FIGURE 6:**
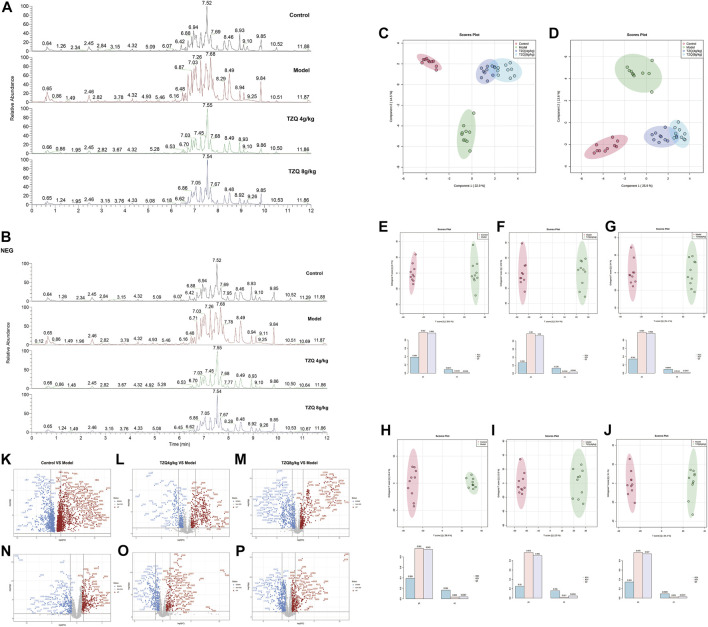
TZQ metabolic biomarker screening in the treatment of atherosclerosis (AS). *n* = 10 mice for each group. **(A)**: Total ion chromatogram of ApoE^−/−^ mouse serum samples in positive ion mode. **(B)**: Total ion current map of ApoE^−/−^ mouse serum samples in negative ion mode. **(C)**: Orthogonal partial least squares analysis (OPLS-DA) score plot of serum samples in positive ion mode. **(D)**: OPLS-DA score map of serum samples in negative ion mode. **(E–G)**: OPLS-DA score plot of serum samples in positive ion mode; **(E)**: Control vs. Model; **(F)**: Model vs. TZQ 4 g/kg; **(G)**: Model vs. TZQ 8 g/kg; **(H–J)**: OPLS-DA score plot of serum samples in negative ion mode. **(H)**: Control vs. Model; **(I)**: Model vs. TZQ 4 g/kg; **(J)**: Model vs. TZQ 8 g/kg. **(K–M)**: Volcano plot of serum samples in positive ion mode; **(K)**: Control vs. Model; **(L)**: Model vs. TZQ 4 g/kg; **(M)**: Model vs. TZQ 8 g/kg. **(N–P)**: Volcano plot of serum samples in negative ion mode; **(N)**: Control vs. Model; **(O)**: Model vs. TZQ 4 g/kg; **(P)**: Model vs. TZQ 8 g/kg.

Compared with those in the Control group, the peak shape and intensity of serum samples in the Model group were substantially different in the positive and negative ion mode, whereas the peak shape and intensity of the TZQ group in positive and negative ion mode were close to those in the Control group (Figures 6A,B). The data were subjected to orthogonal partial least squares analysis (OPLS-DA). In the positive and negative ion mode, the Control group and the Model group showed a good separation (Figures 6C,D), indicating that there were significant differences between the groups, and the model prediction was preferable. The TZQ 4 g/kg group and the TZQ 8 g/kg group were far apart from the Model group and closer to the Control group, indicating that the changes of metabolites between the TZQ group and the Model group were significantly different, the body’s metabolism was clearly disrupted, and TZQ had shown significant improvement in metabolic disorders. Using OPLS-DA, the values of R^2^Y and Q^2^ were used to evaluate the reliability of the model; the model is better if the values are higher. The average values of R^2^Y and Q^2^ in positive ion mode were 0.982 and 0.951 (Figures 6E–G), respectively, whereas in negative ion mode, R^2^Y and Q^2^ had average values of 0.969 and 0.932, respectively (Figures 6H–J). Changes in each metabolite are presented in a volcano plot, with each point representing a different metabolite, red indicating up-regulation, and blue indicating down-regulation. The metabolites exhibit notable differences between the groups in the positive and negative ion mode (Figures 6K–P).

### 3.7 Metabolic biomarkers and metabolic pathway screening of tangzhiqing formula in the treatment of atherosclerosis

**FIGURE 7:**
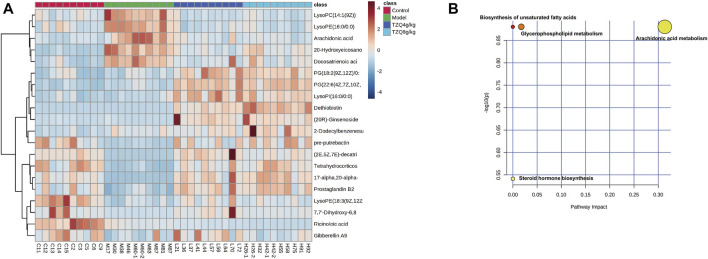
Metabolic biomarkers and metabolic pathway screening of TZQ in the treatment of AS. N = 10 mice for each group. **(A)**: Heatmap of potential biomarkers for the treatment of AS with TZQ. **(B)**: Metabolic pathway analysis.

To better define the differential metabolites with significant variations, the data set range satisfies the conditions of VIP > 1, fold-change>1.2 or <0.8, and *p* < 0.05 as the criteria for screening differential metabolites. A total of 20 potential biomarkers associated with the therapeutic effect of TZQ were identified. Among them, 5 were upregulated and 15 were downregulated. The main components included fatty acids, fatty acyl groups, allyl alcohol lipids, steroids and steroid derivatives, and glycerophospholipids (Figure 7A; Table 2). The metabolic pathways involved in the screened 20 differential metabolites were evaluated, and the metabolic pathways with Impact>0.01 and −log(P) > 0.1 were selected as the metabolic pathways related to the treatment of AS by TZQ. Four metabolic pathways were discovered (Figure 7B; Table 3) and depict arachidonic acid metabolism, glycerophospholipid metabolism, steroid hormone production, and unsaturated fatty acid biosynthesis, respectively.

**TABLE 2:** Potential biomarkers of TZQ in the treatment of atherosclerosis.

Number	RT (min)	m/z	Ion	Formula	Identification	*p*-value	Fold change	Trend
1	3.172	299.25854	[M + H]+	C18H34O3	Ricinoleic acid	1.30E-05	0.411	Down
2	3.777	309.19401	[M + H]+	C17H27NO4	(2E,5Z,7E)-decatrienoylcarnitine	1.84E-12	0.1	Down
3	1.401	316.16745	[M + Na]+	C19H24O4	Gibberellin A9	0.01158	0.5568	Down
4	5.686	350.4923	[M + ACN + H]+	C21H34O4	Tetrahydrocorticosterone	7.93E-06	0.2543	Down
5	6.505	476.27814	[M + H]+	C30H37NO4	LysoPE (18:3 (9Z,12Z,15Z)/0:0)	8.04E-06	0.38609	Down
6	10.129	328.29774	[M−H]−	C20H40O3	20-Hydroxyeicosanoic acid	1.53E-06	2.5102	Up
7	10.121	334.5359	[M−H]−	C22H38O2	Docosatrienoic acid	0.00073885	1.893	Up
8	6.469	452.27823	[M−H]−	C21H44NO7P	LysoPE (16:0/0:0)	1.11E-07	2.0486	Up
9	7.991	305.24823	[M + H]+	C20H34O3	Arachidonic acid	6.57E-06	3.7615	Up
10	5.882	466.29324	[M + H]+	C27H41NO4	LysoPC(14:1 (9Z))	1.99E-05	2.3972	Up
11	3.61	321.03958	[M−H]−	C11H12Cl2N2O5	7,7′-Dihydroxy-6,8′-bicoumarin	0.01893	0.0024111	Down
12	5.555	327.11786	[M + TFA-H]-	C19H20O5	Dethiobiotin	2.76E-09	0.10229	Down
13	7.321	331.22849	[M−H]−	C21H32O3	17-alpha,20-alpha-Dihydroxypregn-4-e	6.70E-06	0.046782	Down
14	6.368	333.20703	[M−H]−	C20H30O4	Prostaglandin B2	3.43E-05	0.47008	Down
15	8.303	507.27365	[M−H]−	C32H36N4O2	PG (18:2 (9Z,12Z)/0:0)	7.12E-05	0.60206	Down
16	8.087	556.28012	[M−H]−	C28H45O9P	PG (22:6 (4Z,7Z,10Z,13Z,16Z,19Z)/0:0)	3.20E-08	0.42825	Down
17	8.327	571.28802	[M−H]−	C25H49O12P	LysoPI(16:0/0:0)	1.87E-05	0.50486	Down
18	11.677	621.43756	[M−H]−	C36H62O8	(20R)-Ginsenoside Rh2	0.0014569	0.080826	Down
19	11.292	325.18491	[M−H]−	C18H30O3S	2-Dodecylbenzenesulfonic acid	0.017177	0.48666	Down
20	8.93	427.15976	[M + K−2H]−	C23H24O8	pre-putrebactin	0.0011465	0.19386	Down

**TABLE 3:** Analysis parameters of metabolic pathways.

Pathway name	Raw p	−log(p)	Holm p	FDR	Impact
Arachidonic acid metabolism	0.13171	0.88038	1.0	1.0	0.3135
Glycerophospholipid metabolism	0.13171	0.88038	1.0	1.0	0.01736
Steroid hormone biosynthesis	0.28749	0.54138	1.0	1.0	1.3E-4
Biosynthesis of unsaturated fatty acids	0.13171	0.88038	1.0	1.0	0

### 3.8 Core genetic screening for the interaction of metabolic biomarkers and pyroptosis

**FIGURE 8:**
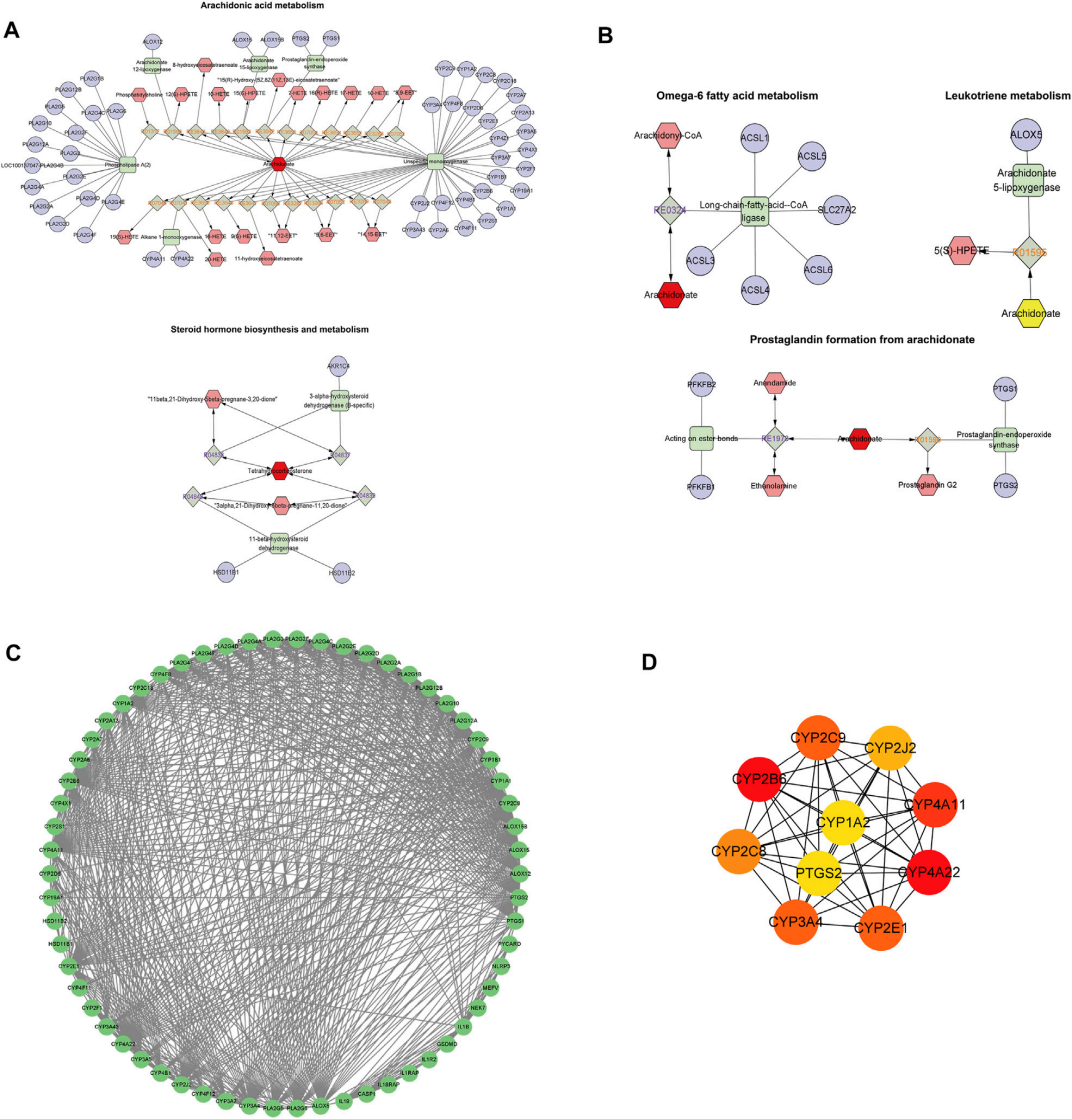
Core genetic screening for the interaction of metabolic biomarkers and pyroptosis. **(A,B)** Compound-Reaction-Enzyme-Gene network day. **(A)**: Arachidonic acid metabolism and steroid hormone biosynthesis and metabolism; **(B)**: Leukotriene metabolism, omega-6 fatty acid metabolism, and prostaglandin formation from arachidonic acid. Green squares: metabolic enzymes; purple circles: genes; red hexagons: metabolites; gray diamonds: enzymatic reactions; orange hexagons: compounds. **(C)**: Protein-protein interaction (PPI) network diagram of 63 metabolite-related genes and 6 pyroptosis-related genes. **(D)**: Core genes. From red to yellow: The darker the color, the greater the degree value and the greater the association.

Data concerning differential metabolites were imported into Cytoscape, and the identities of 20 differential metabolites were imported into the MetScape plug-in to construct a Compound-Reaction-Enzyme-Gene network. The network had a total of 133 nodes, 12 enzymes, 63 genes, 27 compounds, and 31 reactions. It is divided into five sections: arachidonic acid metabolism, steroid hormone biosynthesis and metabolism, leukotriene metabolism, omega-6 fatty acid metabolism, and prostaglandins formed from arachidonic acid (Figures 8A,B). The identities of the 63 metabolite-related genes screened with Compound-Reaction-Enzyme-Gene and 6 related genes (NLRP3, ASC, caspase-1, GSDMD, IL-18, and IL-1β) related to pyroptosis were imported into the STRING database. A protein-protein interaction (PPI) network was constructed (Figure 8C), and the CytoHubba module in Cytoscape software was used to screen the core genes. The top 10 key genes were screened according to the Degree value, including the cytochrome P450 enzyme family (CYP2B6, CYP4A22, CYP4A11, CYP3A4, CYP2E1, CYP2C9, CYP2C8, CYP2J2, and CYP1A2) and cyclooxygenase 2 (COX-2) (Figure 8D).

## 4 Discussion

AS is a disease that primarily affects the intimal layer of the arterial vessel wall. Increased intima-media thickness of the common carotid artery may reflect increased intima thickness, which may be accompanied by an increase in shear and tensile stresses (Glagov et al., 1992). Growing evidence indicates that increased intima-media thickness of the common carotid artery is an indication of AS (Solberg and Eggen, 1971; Polak et al., 2013; Zhang et al., 2017). AS usually begins at the bifurcation and spreads to the proximal common and internal carotid arteries. Furthermore, the velocity of arterial blood flow is linked to the presence of AS plaques. PSV and EDV are not only related to the amount of intra-plaque blood loss, but also to the degree of carotid lumen stenosis. Increased PSV may be an indicator of plaque instability in AS (Mofidi et al., 2005). According to our findings, TZQ considerably lowers the diastolic diameter of the common carotid artery and the systolic blood flow velocity of the aortic arch when compared with that in the Model group. LDL is the principal source of plaque cholesterol in atherosclerotic plaques, which have a lipid-rich core. These cholesterol-rich, like oxidized LDL, areas are accumulated by endothelial cells and absorbed by macrophages, resulting in the formation of foam cells (Talayero and Sacks, 2011). Low HDL-C levels are a significant risk factor for cardiovascular disease and the cardioprotective effects of HDL-C have been attributed to its role in reverse cholesterol transport. Thus, raising HDL-C may help reduce the risk of cardiovascular disease. TZQ and Ator considerably decreased the serum levels of TC, TG, and LDL-C in this investigation while significantly increasing HDL-C. Studies have shown, however, that Ator treatment does not reduce total plasma cholesterol levels nor does it affect diet-induced weight gain (Nachtigal et al., 2006; Bot et al., 2011). Nevertheless, some studies support our findings. The effects of Ator include a significant reduction of total cholesterol levels, lipoproteins (VLDL and LDL), and triglycerides, as well as an increase in HDL levels (Nachtigal et al., 2008). Statistics suggest that statins reduce lipid levels in LDLR^−/−^ mice, but not in ApoE^−/−^ mice (Quarfordt et al., 1995). A study by Sparrow et al. (2001) demonstrated that simvastatin reduced aortic cholesterol accumulation without reducing plasma cholesterol. Observations from these experiments indicate. In ApoE^−/−^ mice, cholesterol levels and atherogenesis may be significantly influenced by the experimental design, including the type of statin administered, the duration of treatment, and the mode of administration. In the present study, TZQ has the potential to minimize aortic plaque formation and collagen content. TZQ alleviated lipid metabolism abnormalities in atherosclerotic mice. AS is a chronic inflammatory disease in which inflammation is involved in all stages of AS. TNF-α is involved in the inflammatory response and affects the balance of blood lipid components. IL-6 possesses a number of features that contribute to the development of cardiovascular disease, as well as a beneficial influence on lipid processing. In addition, IL-6 has the ability to suppress the production of other inflammatory cytokines (Akita et al., 2017). The inflammasome is an innate immunity protein complex that cleaves IL-1β and IL-18 precursors to mature forms and triggers pyroptosis by cleaving caspase-1. Studies have shown that the NLRP3 inflammasome, IL-1β, IL-18, and pyroptosis play important roles in AS (Wu et al., 2018a; He et al., 2021a). Compared with ApoE^−/−^ mice, ApoE^−/−^ and IL-1β^−/−^ mice have approximately 30% fewer atherosclerotic plaques, demonstrating that IL-1β is a key factor in the development of AS (Ling et al., 2008). As shown in clinical research studies, NLRP3 levels in the aorta and ascending aorta are much higher in patients having coronary artery bypass grafting than in individuals without AS (Zheng et al., 2013). Furthermore, the transcript and protein levels of NLRP3, ASC, and caspase-1, as well as IL-1β and IL-18, were shown to be significantly elevated in carotid plaques compared with healthy arteries (Shi et al., 2015). In conclusion, the NLRP3 inflammasome plays a critical role in the development of AS. Among the important proteins, IL-1β and IL-18 are NLRP3 inflammasome activation products, which also play a key role in the formation of AS. In the present study, TZQ substantially reduced the serum concentrations of TNF-ɑ, IL-18, and IL-1β in atherosclerotic mice compared with those in the Model group. TZQ also prevented pyroptosis by reducing the expression of NLRP3, caspase-1, ASC, and GSDMD mRNA and protein levels.

Metabolomics is used to measure the overall metabolic state in cells, tissues, or biological fluids. Because it is downstream of metabolic processes, the metabolome can greatly amplify small functional changes at the genetic or protein expression level (Wishart, 2016). Increasingly, evidence shows that metabolomics can be an effective method for identifying novel biomarkers for AS (Blake et al., 2002). Pyroptosis is followed by inflammasome activation and cytokine release, resulting in inflammatory cell death and an amplification of the inflammatory response. These processes will affect various metabolic disorders in the body. Moreover, the inflammasome is affected by various metabolic risk signals. Cholesterol crystals and oxidized LDL, for example, are examples of activation (He et al., 2021b). Apolipoprotein-M and sphingosine 1-phosphate have been found to reduce AS through inhibiting TNF-α-induced pyroptosis through binding to S1P receptor 2 (Liu and Tie, 2019). Trimethylamine N-oxide, normally produced during phosphatidylcholine metabolism by gut microbiota, enhances ROS-induced pyroptosis of vascular endothelial cells that leads to atherosclerotic lesions development (Wu et al., 2020). Lysophosphatidylcholine, one of the key lipid components composed of ox-LDL and cell membranes, plays critical role in AS. Lysophosphatidylcholine promotes foam cell formation, increases IL-1β secretion, and promotes pyroptosis and lipid core formation (Correa et al., 2019).

We revealed that TZQ regulates 20 atherosclerosis-related metabolites. Further investigation of metabolic pathways revealed that TZQ regulates arachidonic acid metabolism, glycerophospholipid metabolism, steroid hormone biosynthesis, and unsaturated fatty acid biosynthesis. There is, however, a need for further analysis to determine whether these 20 TZQ-regulated metabolites have anti-atherosclerotic properties. The core genes were selected through a correlation analysis of 20 differential metabolite genes and pyroptosis-related genes, and they included several cytochrome P450 enzyme families (CYP2B6, CYP4A22, CYP4A11, CYP3A4, CYP2E1, CYP2C9, CYP2C8, CYP2J2, CYP1A2) and COX-2 (prostaglandin-endoperoxide synthase 2). We want to emphasize, however, that these 10 core genes are only predictions, and their specific relationship with pyroptosis needs further verification in subsequent studies using gene technology such as RT-PCR. Prostaglandin-endoperoxide synthase 2 is a potent pro-inflammatory enzyme commonly known as COX-2. COX-2, the rate-limiting enzyme that catalyzes the transformation of arachidonic acid to prostaglandins, is implicated in a variety of inflammatory reactions triggered by pro-inflammatory cytokines. According to one study, the dephosphorylation of COX-2 enhances the activation of the NLRP3 inflammasome and generates pyroptosis, which releases IL-1β and amplifies the inflammatory response, (Zhang et al., 2019). COX-2 also catalyzes the synthesis of prostaglandin E2 and increases the secretion of IL-1β, and inhibition or knockdown of COX-2 reduces NLRP3 inflammasome activation, IL-1β secretion, and macrophage Pyroptosis (Hua et al., 2015). Cytochrome P450 is an essential enzyme in the metabolism of arachidonic acid, which plays an important role in the occurrence and development of coronary heart disease, diabetes, and other diseases. The cytochrome P450 epoxidase pathway converts arachidonic acid to EET, which possesses vasodilatory, anti-inflammatory, and anti-apoptotic properties. According to one study, EET treatment reduced the expression of NLRP3, caspase-1, and IL-1β, thereby inhibiting the activation of the NLRP3 inflammasome and reducing inflammasome formation and pyroptosis (Zhu et al., 2019). Similarly, EET can regulate NLRP3-induced pyroptosis and alleviate ischemia-reperfusion injury through inhibiting the Toll-like receptor 4 pathway (Zhu et al., 2020). In addition, EET inhibits the activation of the NLRP3 inflammasome by inhibiting calcium overload and ROS generation in macrophages (Luo et al., 2020).

In summary, nine cytochrome P450 enzyme families and COX-2 were evaluated for the link between metabolites and pyroptosis, all of which are involved in the metabolism of arachidonic acid. Therefore, we hypothesized that TZQ inhibits AS via modulating the cytochrome P450 enzyme and the COX-2 metabolic pathway, which ultimately affects pyroptosis. However, there are still many limitations and shortcomings in this study. The non-target metabolomics approach utilized in this work is not sensitive to specific metabolites, and there is no quantitative analysis of the compounds that were screened differentially. Two molecules essential to the metabolism of arachidonic acid (i.e., COX-2 and EET) are closely related to proteins involved in pyroptosis and may promote or inhibit the activation of the NLRP3 inflammasome. The impact of TZQ on COX-2 and EET remains unknown, and more research is required. Furthermore, although this study has shown that TZQ reduces AS through pyroptosis, the precise kind of pyroptosis (endothelial cells, macrophages, or smooth muscle cells) is ambiguous, and far more research is required to improve this situation.

## 5 Conclusion

TZQ attenuated the formation of atherosclerotic lesions and pyroptosis in the aortic intima of high-fat diet ApoE^−/−^ mice. We revealed that TZQ regulates arachidonic acid metabolism, glycerophospholipid metabolism, steroid hormone biosynthesis, and unsaturated fatty acid biosynthesis, all of which have anti-atherosclerotic effects. Moreover, TZQ inhibited AS via modulating the cytochrome P450 enzyme and the COX-2 metaboilic pathway, which ultimately affects pyroptosis. These findings provide a novel mechanism of TZQ in the prevention and reversal of AS.

## Data Availability

The original contributions presented in the study are included in the article/Supplementary Materials, further inquiries can be directed to the corresponding authors.
