# Supplementary figures and images for: Integrated pyroptosis measurement and metabolomics to elucidate the effect and mechanism of tangzhiqing on atherosclerosis
Source: Front Physiol. 2022 Sep 12;13:937737. doi: 10.3389/fphys.2022.937737 (PMC9512057; doi:10.3389/fphys.2022.937737)

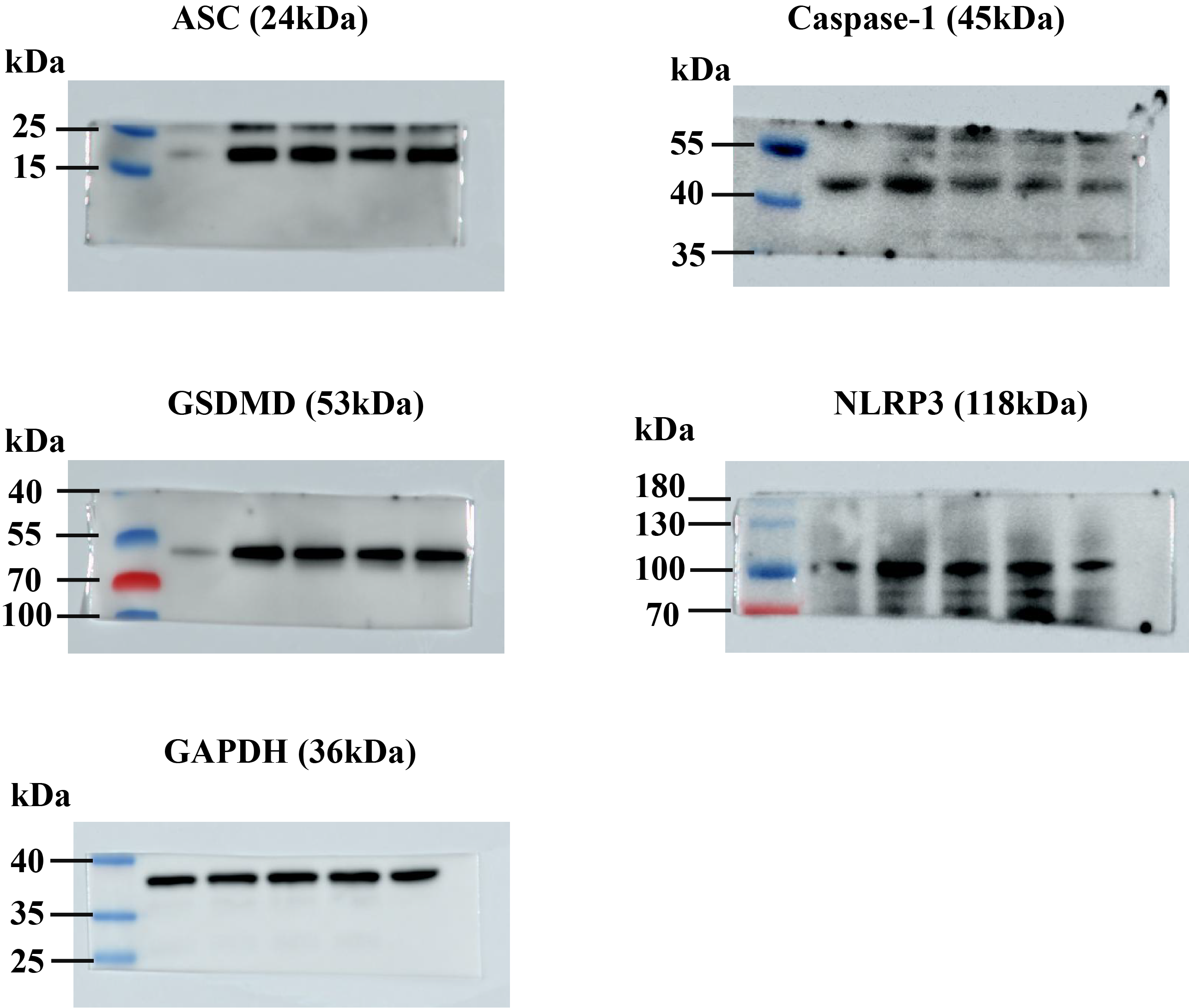

Supplement: Supplementary file 3 [file Image1.TIF]
